# A study of the osmotic characteristics, water permeability, and cryoprotectant permeability of human vaginal immune cells

**DOI:** 10.1016/j.cryobiol.2016.03.003

**Published:** 2016-04

**Authors:** Zhiquan Shu, Sean M. Hughes, Cifeng Fang, Jinghua Huang, Baiwen Fu, Gang Zhao, Michael Fialkow, Gretchen Lentz, Florian Hladik, Dayong Gao

**Affiliations:** a Department of Mechanical Engineering, University of Washington, Seattle, WA 98195, USA; b School of Mechanical and Materials Engineering, Washington State University, Everett, WA 98201, USA; c Department of Obstetrics and Gynecology, University of Washington, Seattle, WA 98195, USA; d Vaccine and Infectious Disease Division, Fred Hutchinson Cancer Research Center, Seattle, WA 98109, USA; e College of Information Technology, Beijing Union University, Beijing 100101, China; f Department of Electronic Science & Technology, University of Science and Technology of China, Hefei 230027, China

**Keywords:** Cryopreservation, Human vaginal mucosa, T cell, Macrophage, Cryobiological characteristics, Microfluidic perfusion channel

## Abstract

Cryopreservation of specimens taken from the genital tract of women is important for studying mucosal immunity during HIV prevention trials. However, it is unclear whether the current, empirically developed cryopreservation procedures for peripheral blood cells are also ideal for genital specimens. The optimal cryopreservation protocol depends on the cryobiological features of the cells. Thus, we obtained tissue specimens from vaginal repair surgeries, isolated and flow cytometry-purified immune cells, and determined fundamental cryobiological characteristics of vaginal CD3^+^ T cells and CD14^+^ macrophages using a microfluidic device. The osmotically inactive volumes of the two cell types (*V*_b_) were determined relative to the initial cell volume (*V*_0_) by exposing the cells to hypotonic and hypertonic saline solutions, evaluating the equilibrium volume, and applying the Boyle van't Hoff relationship. The cell membrane permeability to water (*L*_p_) and to four different cryoprotective agent (CPA) solutions (*P*_s_) at room temperature were also measured. Results indicated *V*_b_ values of 0.516 *V*_0_ and 0.457 *V*_0_ for mucosal T cells and macrophages, respectively. *L*_p_ values at room temperature were 0.196 and 0.295 μm/min/atm for T cells and macrophages, respectively. Both cell types had high *P*_s_ values for the three CPAs, dimethyl sulfoxide (DMSO), propylene glycol (PG) and ethylene glycol (EG) (minimum of 0.418 × 10^−3^ cm/min), but transport of the fourth CPA, glycerol, occurred 50–150 times more slowly. Thus, DMSO, PG, and EG are better options than glycerol in avoiding severe cell volume excursion and osmotic injury during CPA addition and removal for cryopreservation of human vaginal immune cells.

## 1 Introduction

In HIV vaccine and microbicide trials, immune responses are typically evaluated in the peripheral blood, despite the most important immune responses being at the sites of viral entry, namely the genital and rectal mucosae. Sophisticated analyses of fresh mucosal cell and tissue samples are currently being done, but cryopreservation is little used [19], [22], [27]. Cryopreservation of mucosal specimens is critically important for immunological studies because it allows samples obtained at different times and trial sites to be preserved, shipped and stored for later analysis at a central laboratory. However, it is not clear whether the currently-used cryopreservation strategies, which were originally developed for peripheral blood mononuclear cells (PBMC), are ideal for mucosal specimens. Publications reporting functional cell-based assays performed with cryopreserved mucosal specimens are limited and inconsistent [3], [8], [14], [20].

To optimize the cryopreservation of mucosal cells, it is necessary to have a quantitative understanding of their biophysical response to the freezing process [15], [16], [18]. According to Mazur's “Two-Factor Hypothesis”, the cellular response to freezing is governed by intrinsic properties of the cells, including the portion of the cell volume that does not respond to osmotic pressure (*V*_b_), the permeability of the cell membrane to water (*L*_p_) and the permeability of the membrane to cryoprotective agents (CPAs; *P*_s_) [17]. These properties are unknown for mucosal immune cells.

Our hypothesis is that understanding the fundamental cryobiological characteristics of mucosal immune cells will allow the development of an improved cryopreservation procedure. In this work, the cryobiological properties of mucosal immune cells were determined using a microfluidic device developed in our group [1], [2]. Since the female genital tract is one of the most common sites of sexual HIV transmission, the cells assessed were isolated from the human vagina. Specifically, two cell populations that are central to adaptive cellular immunity and HIV susceptibility, T lymphocytes and macrophages, were isolated and their osmotically inactive cell volume (*V*_b_), cell membrane permeability to water (*L*_p_), and cell membrane permeability to CPAs (*P*_s_) were determined. Four widely used CPAs – dimethyl sulfoxide (DMSO), glycerol, propylene glycol (PG) and ethylene glycol (EG) – were tested.

## 2 Materials and methods

### 2.1 Human vaginal mucosal specimens

Human vaginal tissues were obtained from healthy women undergoing vaginal repair surgeries in the Department of Obstetrics and Gynecology at the University of Washington. These tissues, which would otherwise have been discarded, were collected without any identifying patient information under a waiver of consent approved by the Institutional Review Boards of the University of Washington and the Fred Hutchinson Cancer Research Center.

### 2.2 Isolation and sorting of vaginal T cells and macrophages

Vaginal tissues were maintained in saline and on ice during transport and dissection. The stroma was removed from the epithelium, leaving epithelial pieces about 2 mm thick. These were subsequently cut into pieces of about 1 × 1 mm and stored overnight in cell culture medium at 4 °C. The next morning, cells were isolated using an enzymatic digestion protocol [22]. Briefly, tissues were incubated in collagenase type II digestion medium (700 collagen units per mL; Sigma, St. Louis, MO) with 500–1000 units per mL DNase I (Sigma) at 37 °C with shaking for 30 min; tissues were disrupted by passage through a blunt needle and syringe, and the resulting cell suspensions were separated from undigested tissue pieces by filtration through a 70 μm strainer. Remaining tissue pieces were re-digested up to three additional times. Vaginal T cells and macrophages were purified from the bulk cell population by flow cytometric sorting, after staining with CD45 APC, CD3 FITC, and CD14 PE-Cy7 (all mouse anti-human from BD Biosciences, San Jose, CA, USA) and 0.1 μg/mL 4′,6-diamidino-2-phenylindole (DAPI) for viability. All antibodies were titrated before use and used at the minimum saturating dose. Live CD45^+^CD3^+^CD14^−^ and live CD45^+^CD3^−^CD14^+^ events were sorted on a four laser BD FACSAria II (408, 488, 535, and 633 nm). The sorted cells were suspended in 1× PBS at 10,000 cells/mL, stored at 4 °C, and used for the following experiments within 8 h.

### 2.3 The microfluidic perfusion system

Cell membrane permeabilities were measured with a microfluidic perfusion chamber we developed previously [1]. The microfluidic device was fabricated using soft lithography. The height of the microfluidic perfusion chamber was 15 μm to accommodate a monolayer of the expected cell sizes (8–12 μm). At the edge of the chamber, the channel height was shortened to 3 μm to trap the cells but still allow fluid to flow.

During experiments, the microfluidic device was immobilized on the stage of the microscopy (DM IRB, Leica, Buffalo Grove, IL). A droplet of cell suspension (∼10 μL) was added gently to the inlet reservoir. The fluid was withdrawn continuously by a digitally controlled syringe pump (PHD 2000 Infusion, Harvard Apparatus, Holliston, MA) with a flow rate of 40 μL/h in order to stably trap cells in the chamber. After 10–15 cells were trapped and aligned in front of the block, 0.5 mL perfusion solution was added into the inlet reservoir, avoiding any violent perturbation to the fluid flow. The fluid was drawn into the chamber continuously by the syringe pump. The cell volume excursion history was recorded by a CCD camera (Phantom v310, Vision Research, Wayne, NJ) at 24 frames/second until osmotic equilibrium was obtained, generally within 2 min. All the experiments were done at room temperature (∼22 °C).

In order to measure the osmotically inactive cell volume (*V*_b_) and the cell membrane permeability to water (*L*_p_), trapped cells were perfused with hypotonic and hypertonic saline solutions (0.7× PBS, 2× PBS and 3× PBS). To determine the cell membrane permeability to DMSO, glycerol, PG, and EG, dilutions of these chemicals were prepared in 0.9% NaCl saline solution. The osmolalities of the solutions were measured by an osmometer (Wescor Inc., Logan, UT) based on vapor pressure assessment (Table 1).

### 2.4 Image analysis

**Fig. 1:**
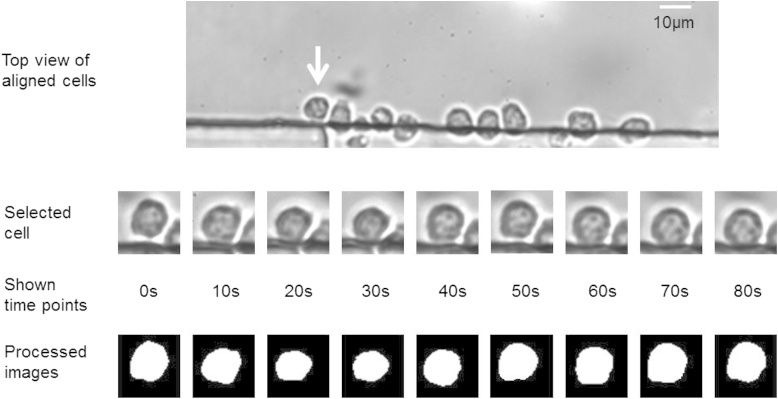
Cell volume excursion during perfusion and image processing. T cells were perfused by 10% DMSO in 0.9% NaCl.

After video capture, the videos were converted to image frames using Cine Viewer software (Vision Research, Wayne, NJ). Cells were cropped from each frame of the image. The cropped images were enhanced and processed to find the cell boundary (see Fig. 1). In order to detect the cell boundary precisely, the “Active Contour (dual-snake)” algorithm was applied [7]. Thereafter, the two-dimensional cell area was evaluated by pixel counting and then converted to three-dimensional cell surface area and volume based on the assumption of spherical cell shape. All the image processing was performed with MATLAB software (MathWorks, Natick, MA).

In order to assess the hypothesis of spherical cell shape, the sphericity of T cells and macrophages (cell images at the beginning of each experiment) was evaluated, which was defined as 2π·requpact. Here, *r*_*equ*_ is the equivalent cell radius calculated with the two-dimensional cell area based on image analysis, and *p*_*act*_ is the actual cell perimeter.

### 2.5 Determination of osmotically inactive cell volume (*V*_*b*_)

*V*_b_, the osmotically inactive volume of the cell (μm^3^), can be determined by the Boyle van't Hoff relationship. Assuming the cell acts as an ideal osmometer, the osmotic response of the cell volume during hypertonic shrinkage can be described as

(1) $$V=\frac{C_{0}\left(V_{0}-V_{b}\right)}{C_{n}^{i}}+V_{b},$$

where V (μm^3^) is the cell volume when the intracellular osmolality is $C_{n}^{i}$ (Osm/kg water), *V*_0_ is the isotonic cell volume, *C*_0_ is the isotonic osmolality, and *V*_b_ is the osmotically inactive cell volume.

### 2.6 Determination of cell membrane permeability to water (*L*_*p*_) when no CPA exists

The membrane permeabilities to water (*L*_p_) of human vaginal mucosal immune cells (T cells and macrophages) were determined by measuring cell volume shrinkage while cells were perfused by hypertonic 2× or 3× PBS solutions. The cell volume change, i.e., water transport across the cell membrane, can be described as [1], [5], [21]

(2) $$\frac{{\text{dV}}_{\text{c}}\left(\text{t}\right)}{\text{dt}}={\text{L}}_{\text{p}}\cdot \text{A}\cdot \left({\text{C}}_{\text{n}}^{\text{i}}-{\text{C}}_{\text{n}}^{\text{e}}\right)\cdot \text{R}\cdot \text{T}\text{,}$$

where ***V***_***c***_(***t***) is the cell volume (μm^3^) at time t (min); ***L***_**p**_ is the cell membrane permeability to water (μm/atm/min); A is the cell membrane area (μm^2^) and assumed as constant during perfusion (=4**πr**^2^ for a spherical cell shape); $C_{n}^{i},C_{n}^{e}$ are the intracellular and extracellular molalities (Osm/kg water), respectively; R is the universal gas constant (=0.08207 (atm L)/(mol K); and T is absolute temperature (in Kelvin). It is assumed that the cells are spherical. The ***L***_**p**_ was determined by least-squares curve fitting of the cell volume change data to the equation using MLAB (Civilized Software Inc., Silver Spring, MD).

### 2.7 Determination of cell membrane permeabilities to water (*L*_*p*_) and CPA (*P*_*s*_): two-parameter transport formalism

When permeant CPA (e.g., DMSO) and salts (e.g., NaCl) co-exist in a solution, the cell membrane permeability to water (*L*_p_) and to the CPA (*P*_s_) can be determined with a two-parameter transport model, where the cell volume change depends on both factors [1], [2], [11], [12], [25]:

(3) $$\frac{{\text{dV}}_{\text{c}}\left(\text{t}\right)}{\text{dt}}=\frac{{\text{dV}}_{\text{s}}\left(\text{t}\right)}{\text{dt}}+{\text{L}}_{\text{p}}\cdot \text{A}\cdot \left({\text{C}}^{\text{i}}-{\text{C}}^{\text{e}}\right)\cdot \text{R}\cdot \text{T,}$$

where V_c_(t) and V_s_(t) are cell volume and intracellular CPA volume, respectively, at time t, and C^i^ and C^e^ are intra- and extracellular molalities (including both salts and CPA).

The CPA flux is given by

(4) $$\frac{{\text{dN}}_{\text{s}}\left(\text{t}\right)}{\text{dt}}={\text{P}}_{\text{s}}\cdot \text{A}\cdot \left({\text{C}}_{\text{s}}^{\text{e}}-{\text{C}}_{\text{s}}^{\text{i}}\right)\text{,}$$

where *P*_s_ is the cell membrane permeability to the CPA (cm/min); ${\text{C}}_{\text{s}}^{\text{e}}\;\text{and}\;{\text{C}}_{\text{s}}^{\text{i}}$ are the extracellular and intracellular CPA molalities, respectively; and *N*_s_(*t*) is the mole of intracellular CPA at time t.

*N*_s_(*t*) and *V*_s_(*t*) are interchangeable by

(5) $${\text{N}}_{\text{s}}\left(\text{t}\right)={\text{V}}_{\text{s}}\left(\text{t}\right)/{\bar{\text{V}}}_{\text{s}}.$$

Here, $\bar{V_{\text{s}}}$ is the partial molar volume of the CPA.

The determination of *L*_p_ and *P*_s_ was done by least-squares curve fitting of the experimental data to the above two-parameter formalism using MLAB (Civilized Software Inc.).

### 2.8 CPA exposure tolerance

The CPA exposure tolerance of human vaginal T cells and macrophages to DMSO, EG and PG was tested. CPA solutions with different concentrations were prepared and precooled to 4 °C. 100 μL of each CPA solution was added dropwise to 100 μL cell suspension with agitation over 5 min. The final CPA concentrations ranged from 5% to 17.5% (v/v). After CPA addition, the cell suspension was kept at 4 °C for 10 min. Then, CPA was removed by adding 4 mL isotonic PBS dropwise with agitation at 4 °C over 5 min. The cells were collected by centrifugation at 300*g* for 10 min, and then tested for cell viability with flow cytometry.

### 2.9 Statistical analysis

The number of data sets for the investigation of each cell property (e.g., the membrane permeability to DMSO for T cells) was 7–15 cells total per CPA and cell type from 4 donors. The statistical analysis was performed using the Student's *t-*test. The results are presented as mean ± standard deviation and a P-value less than 0.05 was considered statistically significant.

## 3 Results

### 3.1 Osmotically inactive cell volume *V*_*b*_

**Fig. 2:**
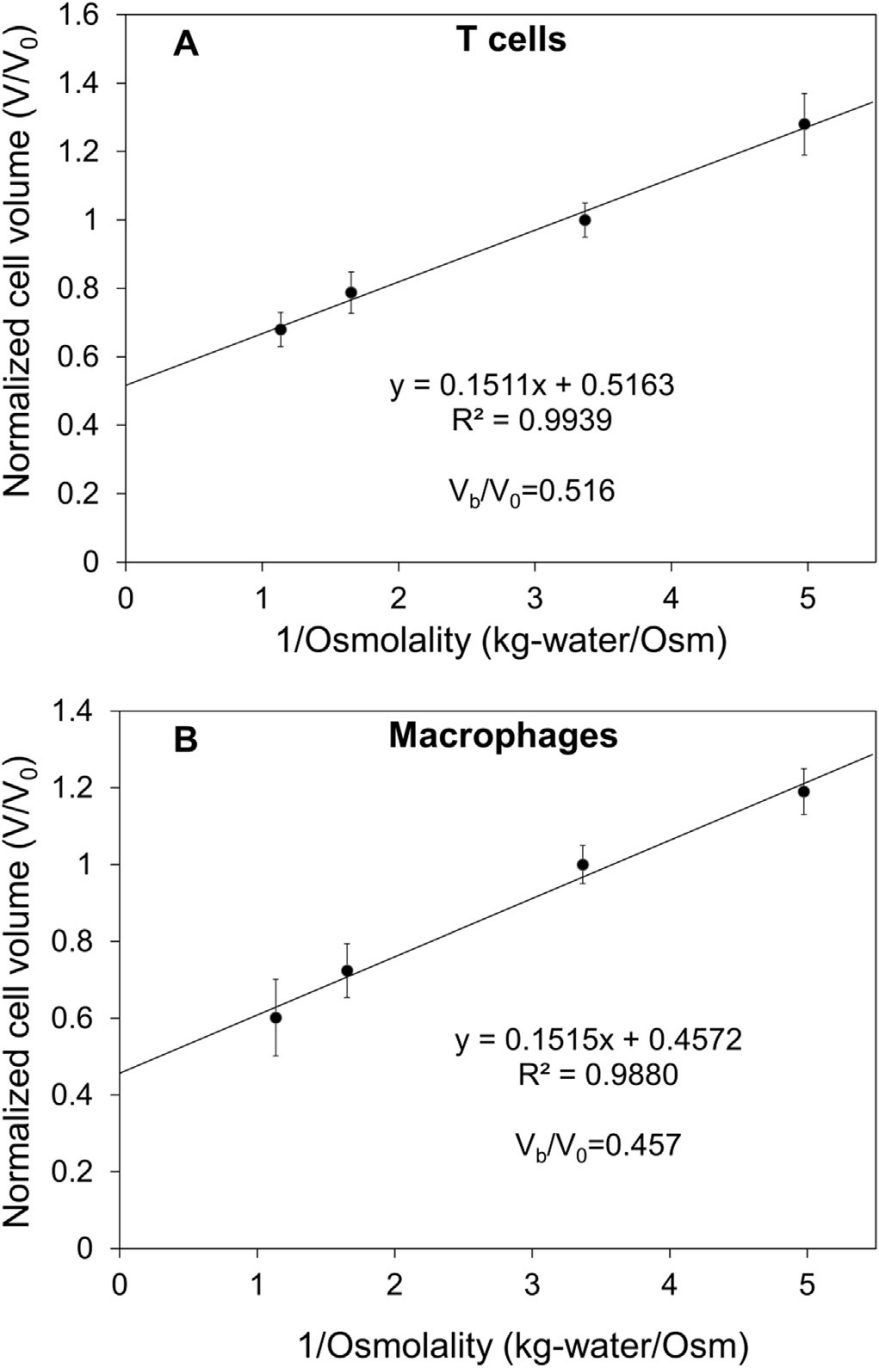
Determination of the osmotically inactive cell volume ***V***_**b**_ for human vaginal mucosal immune cells. Results are presented as mean ± standard deviation (7–8 cells from 4 donors for each data point). (A) Linear curve fitting for T cells. (B) Linear curve fitting for macrophages.

The Boyle van't Hoff plots of human vaginal mucosal T cells and macrophages are shown in Fig. 2. The equilibrium cell volumes in hypotonic and hypertonic saline solutions (0.7×, 2× and 3× PBS) normalized to the cell volume in isotonic solution are plotted with respect to the reciprocal of the osmolality of the solution. The y-intercept is the osmotically inactive cell volume fraction ($V_{b}/V_{0}$), i.e., the remaining cell volume when the osmolality approaches infinity. Results showed that the cell volumes in isosmotic solution (*V*_0_) were 314.61 ± 36.45 μm^3^ and 467.12 ± 32.71 μm^3^ with diameters of 8.43 + 0.32 μm and 9.62 ± 0.23 μm for T cells and macrophages, respectively. The osmotically inactive volumes *V*_b_ of T cells and macrophages were determined to be 51.6% *V*_0_ and 45.7% *V*_0_, respectively.

### 3.2 Cell membrane permeabilities to water (*L*_*p*_) and cryoprotective agents (*P*_*s*_)

**Fig. 3:**
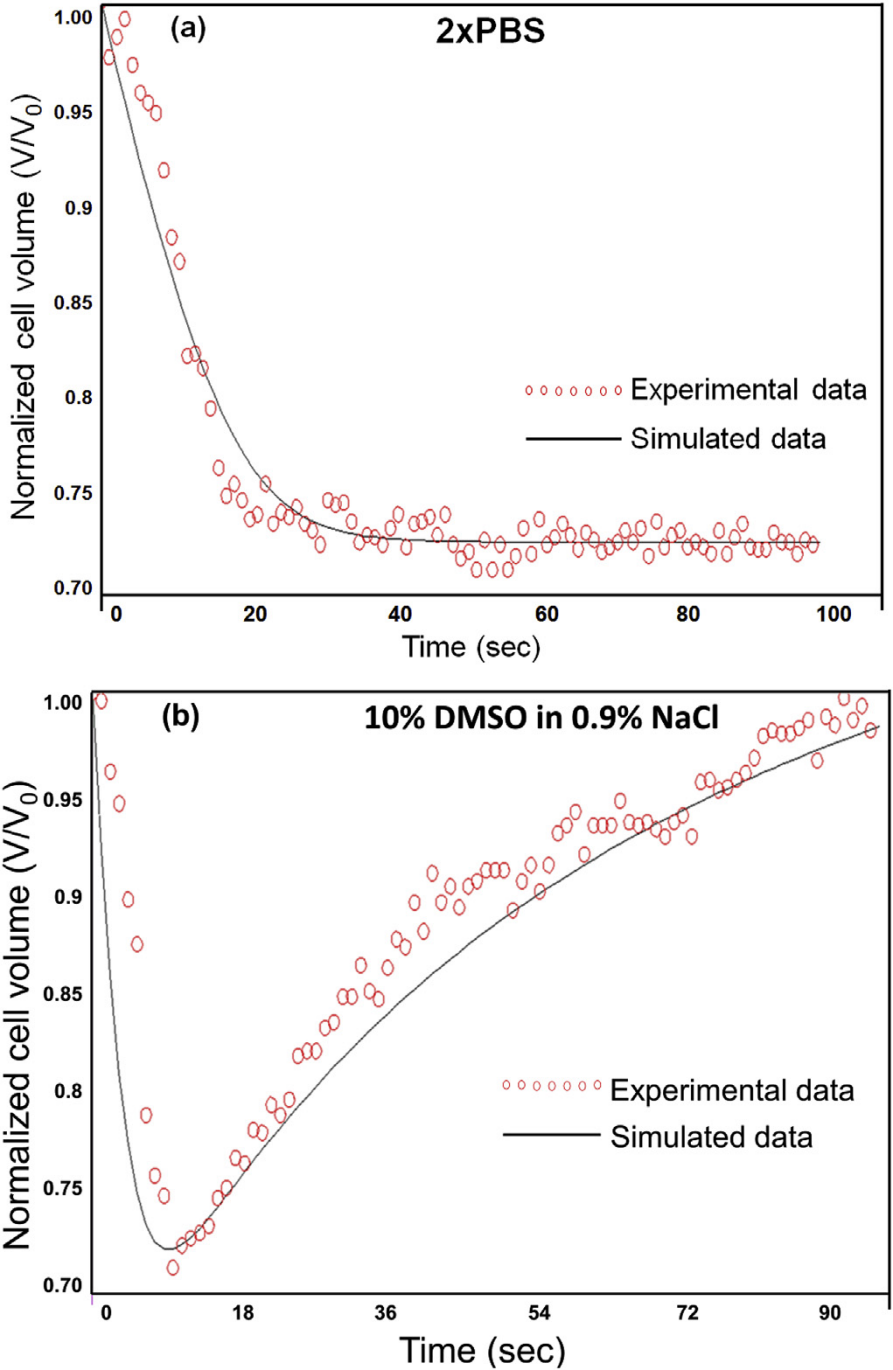
Cell volume excursion during perfusion by hypertonic solutions. (a) T cell volume excursion when perfused by a hypertonic saline solution (2× PBS). (b) T cell volume excursion when perfused by a hypertonic CPA solution (10% DMSO in 0.9% NaCl).

Examples of the T cell volume excursion history when perfused by a hypertonic saline solution and a permeant CPA solution are shown in Fig. 3(a) and Fig. 3(b), respectively. The cell volume derived from the last of the 24 frames in each second was calculated and presented in the figures.

Fig. 3-a shows that when a cell is exposed to a hypertonic saline solution, its volume monotonically decreases and then reaches the final equilibrium value. Based on these data, the water transport ability, i.e., cell membrane permeability to water *L*_*p*_, can be simulated. Fig. 3(b) shows the volume excursion of one cell perfused by 10% DMSO in 0.9% NaCl solution. The result shows that the cell shrinks first and then expands gradually back to a volume close to the original isotonic one. This phenomenon is caused by the transport of both water and permeant CPA. According to the cell volume excursion history, the cell membrane permeabilities to water and CPA can be calculated.

The cell membrane permeabilities to water (L_p_) and CPA (P_s_) were simulated by least-squares curve fitting using MLAB software. The results are shown in Table 2 and Table 3 for human vaginal mucosal T cells and macrophages, respectively. L_p_ values for T cells and macrophages were 0.196 ± 0.047 and 0.295 ± 0.069 μm/min/atm (mean ± standard deviation), respectively, when no CPA exists. If CPA and salts coexist in the solution, L_p_ values were reduced, especially for T cells (p < 0.05). In order to test the assumption that cells are spherical, the sphericity of cells (the cell images at the beginning of each experiment) was evaluated. The sphericities were determined to be 0.91 ± 0.04 (n = 45) for T cells, and 0.88 ± 0.04 (n = 48) for macrophages. The imperfect spherical cell shape may cause errors to the data analysis. However, quantitative evaluation of the effect of non-spherical cell shape on the results is complicated and out of the scope of this work.

Glycerol showed very low *P*_s_ values for both T cells (0.005 ± 0.004 × 10^−3^ cm/min) and macrophages (0.008 ± 0.003 × 10^−3^ cm/min). This was 52–146 times lower than the P_s_ values measured for the other three CPAs (p < 0.05). For T cells, the *P*_s_ values for ethylene glycol, propylene glycol, and DMSO ranged between 0.469 and 0.635 × 10^−3^ cm/min, and there was no statistical evidence of a difference between them (p = 0.465–0.493). For macrophages, *P*_s_ to ethylene glycol (0.418 ± 0.074 × 10^−3^ cm/min) was in the same range as the *P*_s_ values for T cells, but *P*_s_ values for DMSO (0.978 ± 0.313 × 10^−3^ cm/min) and propylene glycol (1.168 ± 0.484 × 10^−3^ cm/min) were significantly higher than the values for T cells (p < 0.05).

### 3.3 CPA exposure tolerance

**Fig. 4:**
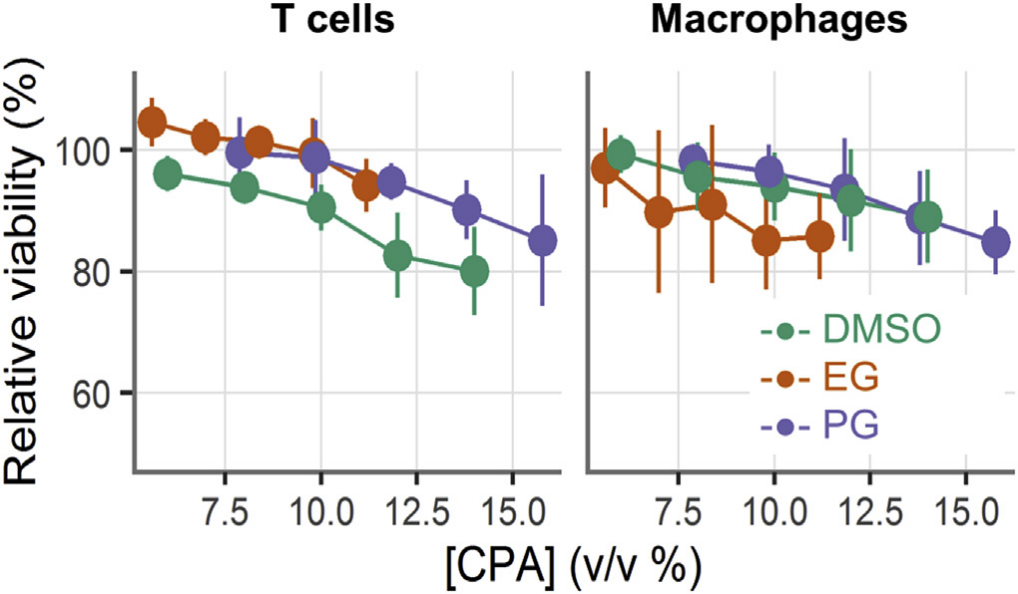
CPA exposure tolerance tests for human vaginal mucosal T cells and macrophages. The x-axis shows the final CPA concentration (v/v, %) after addition into the cell suspensions. The cell viabilities were tested with flow cytometry after CPA addition and removal, and the results were normalized to the viability of fresh cells (mean ± STD, n = 4–5).

To determine the CPA exposure tolerance of mucosal cells, we added DMSO, EG, or PG at various concentrations dropwise to cell suspensions, incubated for 10 min on ice, and then dropwise diluted them out. We measured viability by cell type with flow cytometry, normalizing to the viability of untreated cells to have a consistent measure across samples. Fig. 4 shows the CPA exposure tolerance for human vaginal mucosal T cells and macrophages. The cell viability declined in a linear fashion as CPA concentrations increased. The relative viabilities remained above 90% up to concentrations of about 10% (v/v). DMSO had a more negative effect on T cell viability than the other two CPAs, while EG had a more negative effect on macrophage viability than the other two.

It is worth noting that three permeating CPAs (DMSO, EG and PG) were tested in the CPA exposure tolerance experiment due to their possible applications in the cryopreservation of mucosal cells. Osmotic tolerance limit (OTL) of the cell is another important cryobiological characteristic. For OTL test, cells are exposed to hypo- and hyperosmotic solutions with varying concentrations of non-permeating solute (e.g., NaCl), and then restored to osmotic conditions [28]. Besides the cell viability, cell volume excursion data in this process are also valuable. The obtained cell shrinkage and swelling limits are useful to optimize the protocols of addition and removal of both permeating and non-permeating CPAs. OTL tests of mucosal cells will be done in the future.

## 4 Discussion

Cryopreservation of mucosal immune cells or tissues is essential to evaluate HIV vaccines and microbicides. However, there have been few reports of successful mucosal cryopreservation so far. We believe that this is due to a lack of knowledge about the cryobiological characteristics of such specimens. Based on Mazur's theory, freezing of living cells is a process of heat and mass transfer. The cell type-dependent optimal cryopreservation protocol is determined by the intrinsic biophysical properties of each cell type. Therefore, optimization of mucosal cell cryopreservation requires knowing the specimen's properties, such as the osmotically inactive cellular volume and cell membrane permeabilities to water and to CPAs.

In this work, we used a microfluidic perfusion device to measure the cryobiological properties of human vaginal T cells and macrophages. Table 4 shows the values of these properties for other cell types. It shows that human vaginal immune cells have lower *L*_p_ values than oocytes, prostate cancer cells, and megakaryocyte cells, and similar *L*_p_ values to those of pancreatic islets and dendritic cells. This suggests that CPA addition and removal should be relatively slow to decrease osmotic injury to mucosal immune cells. This might also indicate that water transfers relatively slowly across cell membranes when T cells and macrophages are frozen (measurement of *L*_p_ at subzero temperatures is needed to confirm this), and therefore mucosal immune cells should be frozen at a relatively low cooling rate. Results also showed that *L*_p_ values are reduced in the presence of CPA. Further cryopreservation experiments are necessary to optimize the protocol.

Among the four types of CPAs measured, glycerol had much lower permeability than the other three CPAs, for both T cells and macrophages. Therefore, glycerol can cause severe cell volume excursion and osmotic injury during CPA addition and removal, and is thus the worst option for vaginal immune cell cryopreservation. The *P*_s_ values for ethylene glycol, propylene glycol, and DMSO were similar for T cells, while for macrophages *P*_s_ to DMSO and propylene glycol was two to three times of that for ethylene glycol. For both T cells and macrophages, there was no significant difference between the *P*_s_ values for DMSO and propylene glycol. Tests of cytotoxicity and cryopreservation of mucosal immune cells had similar results for DMSO, ethylene glycol, and propylene glycol (separate manuscript in preparation). Therefore, these three CPAs are likely better options than glycerol for cryopreservation of mucosal T cells and macrophages. Currently, the cryopreservation protocol of human vaginal immune cells is generally adopted from that for peripheral blood mononuclear cell (PBMC), where 10% DMSO and cooling rate of 1 °C/min are applied. Our data necessitate further experimental trials with DMSO, ethylene glycol, propylene glycol, or cocktail of them.

The microfluidic perfusion method used here has some limitations. As a photomicrographic method, its measurement accuracy depends on the quality of captured images and accuracy of image processing. It is applicable to only spherical cells because that is assumed in the conversion from two-dimensional image to three-dimensional volume. The measured result is not the average of many cells, but of a few individual cells. Although it can be used to measure cell membrane properties at other supra-zero temperatures (e.g., 10 °C, 4 °C) if a temperature controller is integrated with the microfluidic device [25], it cannot be applied at sub-zero temperatures due to liquid freezing in the channel. The cell membrane properties at sub-zero temperatures during cooling, especially in the range 0 to −35 °C, are critical for the theoretical prediction of the optimal cooling protocol. A method based on thermal analysis of the cell suspension during freezing using differential scanning calorimetry (DSC) can be used to investigate the cellular biophysical properties at sub-zero temperatures [4]. DSC measurements with purified vaginal T cells and macrophages are currently ongoing in our laboratory.

## 5 Conclusions

In this work, a microfluidic perfusion channel was applied to measure the cryobiological characteristics of human vaginal mucosal T cells and macrophages at room temperature. The osmotically inactive volumes for T cell and macrophage are 0.516 *V*_0_ and 0.457 *V*_0_. Membrane permeabilities to water(*L*_p_) at room temperature for T cells and macrophages are 0.196 and 0.295 μm/min/atm, respectively when no CPA exists in the solution. Among the four tested CPAs, DMSO, ethylene glycol, and propylene glycol have 50–150 times higher *P*_s_ values than glycerol. These three CPAs may be better CPA options to avoid severe cell volume excursion and osmotic injury during CPA addition/removal and cryopreservation. CPA exposure tolerance tests showed that the relative viabilities remained above 90% up to concentrations of about 10% (v/v). DMSO had a more negative effect on T cell viability than the other two CPAs, while EG had a more negative effect on macrophage viability than the other two. In the future, more experiments are needed to further compare the CPAs and optimize the cryopreservation protocol for these cells.

## Figures and Tables

**Table 1:** Perfusion solutions and osmolalities.

Perfusion solutions	Osmolality (mOsm/kg-H_2_O)
0.7× PBS	201
1× PBS	297
2× PBS	605
3× PBS	881
10% (v/v) DMSO in 0.9% NaCl	1823
1.5 M glycerol in 0.9% NaCl	1956
1.5 M ethylene glycol in 0.9% NaCl	1761
1.5 M propylene glycol in 0.9% NaCl	1575

**Table 2:** Membrane permeabilities of T cells to water and CPAs at room temperature (mean ± standard deviation).

CPA	Cells	Lp (μm/min/atm)	Ps(10^−3^ cm/min)
PBS	14	0.196 ± 0.047	
DMSO	8	0.089 ± 0.051	0.472 ± 0.230
Propylene glycol	8	0.077 ± 0.054	0.635 ± 0.342
Ethylene glycol	7	0.099 ± 0.053	0.469 ± 0.175
Glycerol	8	0.055 ± 0.003	0.005 ± 0.004

**Table 3:** Membrane permeabilities of macrophages to water and CPAs at room temperature (mean ± standard deviation).

CPA	Cells	*L*p (μm/min/atm)	*P*s(10^−3^ cm/min)
PBS	15	0.295 ± 0.069	
DMSO	9	0.234 ± 0.041	0.978 ± 0.313
Propylene glycol	9	0.221 ± 0.162	1.168 ± 0.484
Ethylene glycol	8	0.241 ± 0.094	0.418 ± 0.074
Glycerol	7	0.192 ± 0.072	0.008 ± 0.003

**Table 4:** ***L***_**p**_ and ***P***_**s**_ (to DMSO) of some cell types at room temperature (RT).

Cell type	*L*_p_ at RT (μm/min/atm)	*P*_s_ to DMSO at RT (10^−3^ cm/min)	Reference
Rat basophilic leukemia cell	0.38	0.49	[1]
Mouse ovum	0.43		[13]
Mouse oocyte	0.48		[10]
Mouse oocyte	0.45		[6]
Golden hamster pancreatic islet	0.27		[5]
Human prostate cancer cell	0.45		[24]
Mouse dendritic cell	0.17	0.63	[2]
Human megakaryocyte cell	2.26	1.8	[25]
Human granulocyte	0.18	0.64	[26]
Human lymphocyte (from blood)	0.46		[9]
Human lymphocyte (from blood)	0.188		[23]
Human vaginal mucosal T cell	0.196	0.472	Current study
Human vaginal mucosal macrophage	0.295	0.978	Current study

## References

[1] Chen H., Purtteman J.J.P., Heimfeld S., Folch A., Gao D. (2007). Development of a microfluidic device for determination of cell osmotic behavior and membrane transport properties. Cryobiology.

[2] Chen H., Shen H., Heimfeld S., Tran K.K., Reems J., Folch A., Gao D. (2008). A microfluidic study of mouse dendritic cell membrane transport properties of water and cryoprotectants. Int. J. Heat Mass Transf..

[3] Cohen C., Cohen C., Moscicki A., Scott M.E., Shiboski S., Bukusi E., Daud I., Rebhapragada A., Brown J., Kaul R. (2010). Increased levels of immune activation in the genital tract of healthy young women from sub-Saharan Africa. AIDS.

[4] Devireddy Ramachandra V., Raha Debopam, Bischof John C. (1998). Measurement of water transport during freezing in cell suspensions using a differential scanning calorimeter. Cryobiology.

[5] Gao D.Y., Benson C.T., Liu C., McGrath J.J., Critser E.S., Critser J.K. (1996). Development of a novel microperfusion chamber for determination of cell membrane transport properties. Biophys. J..

[6] Gao D., Mcgrath J., Tao J., Benson C., Critser E., Critser J. (1994). Membrane-transport properties of mammalian oocytes – a micropipette perfusion technique. J. Reprod. Fertil..

[7] Gunn S.R., Nixon M.S. (1997). A robust snake implementation; a dual active contour. Pattern Anal. Mach. Intell. IEEE Trans..

[8] Gupta P., Ratner D., Patterson B.K., Kulka K., Rohan L.C., Parniak M.A., Isaacs C.E., Hillier S. (2006). Use of frozen-thawed cervical tissues in the organ culture system to measure anti-HIV activities of candidate microbicides. AIDS Res. Hum. Retrovir..

[9] Hempling H.G., Thompson S., Dupre A. (1977). Osmotic properties of human lymphocyte. J. Cell. Physiol..

[10] Hunter J., Bernard A., Fuller B., Mcgrath J., Shaw R. (1992). Measurements of the membrane water permeability (Lp) and its temperature-dependence (activation-energy) in human fresh and failed-to-fertilize oocytes and mouse oocyte. Cryobiology.

[11] Jacobs M.H. (1933). The simultaneous measurement of cell permeability to water and to dissolved substances. J. Cell. Comp. Physiol..

[12] Kleinhans F.W. (1998). Membrane permeability modeling: Kedem-Katchalsky vs a two-parameter formalism. Cryobiology.

[13] Leibo S.P. (1980). Water permeability and its activation energy of fertilized and unfertilized mouse ova. J. Membr. Biol..

[14] Liebenberg L.J., Gamieldien H., Mkhize N.N., Jaumdally S.Z., Gumbi P.P., Denny L., Passmore J.S. (2011). Stability and transport of cervical cytobrushes for isolation of mononuclear cells from the female genital tract. J. Immunol. Methods.

[15] Mazur P. (1990). Equilibrium, quasi-equilibrium, and nonequilibrium freezing of mammalian embryos. Cell Biophys..

[16] Mazur P. (1984). Freezing of living cells: mechanisms and implications. Am. J. Physiol..

[17] Mazur P. (1963). Kinetics of water loss from cells at subzero temperatures and the likelihood of intracellular freezing. J. General Physiol..

[18] Mazur P. (1970). Cryobiology: the freezing of biological systems. Science.

[19] McElrath M.J., Haynes B.F. (2010). Induction of immunity to human immunodeficiency virus type-1 by vaccination. Immunity.

[20] McGowan I., Tanner K., Elliott J., Ibarrondo J., Khanukhova E., McDonald C., Saunders T., Zhou Y., Anton P.A. (2012). Nonreproducibility of “snap-frozen” rectal biopsies for later use in ex vivo explant infectibility studies. AIDS Res. Hum. Retrovir..

[21] McGrath J.J. (1997). Quantitative measurement of cell membrane transport: technology and applications. Cryobiology.

[22] Mckinnon L., Hughes S., Rosa S., Martinson J., Plants J., Brady K., Gumbi P., Adams D., Vojtech L., Galloway C., Fialkow M., Lentz G., Gao D., Shu Z., Nyanga B., Izulla P., Kimani J., Kimwaki S., Bere A., Moodie Z., Landay A., Passmore J., Kaul R., Novak R., Mcelrath M., Hladik F. (2014). Optimizing viable leukocyte sampling from the female genital tract for clinical trials: an international multi-site study. PLoS One.

[23] Porsche A.M., Körber C., Englich S., Hartmann U., Rau G. (1986). Determination of the permeability of human lymphocytes with a microscope diffusion chamber. Cryobiology.

[24] Takamatsu H., Komori Y., Zawlodzka S., Fujii M. (2004). Quantitative examination of a perfusion microscope for the study of osmotic response of cells. J. Biomech. Eng. Trans. ASME.

[25] Tseng H., Sun S., Shu Z., Ding W., Reems J., Gao D. (2011). A microfluidic study of megakaryocytes membrane transport properties to water and dimethyl sulfoxide at suprazero and subzero temperatures. Biopreserv. Biobank..

[26] Vian A.M., Higgins A.Z. (2014). Membrane permeability of the human granulocyte to water, dimethyl sulfoxide, glycerol, propylene glycol and ethylene glycol. Cryobiology.

[27] Virgin H.W., Walker B. (2010). Immunology and the elusive AIDS vaccine. Nature.

[28] Willoughby C.E., Mazur P., Peter A.T., Critser J.K. (1996). Osmotic tolerance limits and properties of murine spermatozoa. Biol. Reprod..

